# Beyond immune recovery: Quality of life and psychosocial challenges in children with inborn errors of immunity after transplantation

**DOI:** 10.1007/s11845-026-04480-2

**Published:** 2026-06-13

**Authors:** Sezin Aydemir, Ekin Nurhan, Isilay Turan, Necmiye Keser Ozturk, Betul Gemici Karaaslan, Zeynep Meric, Selami Ulas, Sevgi Bilgic Eltan, Haluk Cezmi Cokuğras, Elif Karakoç Aydıner, Safa Baris, Ahmet Ozen, Cigdem Aydogmus, Esra Yucel, Gizem Durcan, Mesut Yavuz, Burak Dogangun, Ayca Kiykim

**Affiliations:** 1 Cerrahpasa Faculty of Medicine, Department of Pediatric Allergy and Immunology, Istanbul University-Cerrahpasa, Kocamustafapasa, Fatih, Istanbul, 34098 Türkiye; 2 Cerrahpasa Faculty of Medicine, Department of Pediatrics, Istanbul University-Cerrahpasa, Istanbul, Türkiye; 3 Department of Pediatric Allergy and Immunology, Basaksehir Cam and Sakura City Hospital, Istanbul, Türkiye; 4 Faculty of Medicine, Department of Pediatrics, Division of Allergy and Immunology, Immune Deficiency Research and Application Center, Marmara University Hospital, Istanbul Jeffrey Modell Diagnostic and Research Center for Primary Immunodeficiencies, The Isil Berat Barlan Center for Translational Medicine, Istanbul, Türkiye; 5 Cerrahpasa Faculty of Medicine, Department of Child and Adolescent Psychiatry, Istanbul University-Cerrahpasa, Istanbul, Türkiye

**Keywords:** Anxiety, Children, Hematopoietic stem cell transplantation, Inborn errors of immunity, Quality of life

## Abstract

**Introduction:**

Hematopoietic stem cell transplantation (HSCT) provides definitive treatment for many inborn errors of immunity (IEI). Although most children achieve full immune reconstitution, long-term psychosocial and quality-of-life outcomes in medically stable survivors remain insufficiently understood.

**Methods:**

Children aged 8–18 years with IEI who achieved complete immune recovery and had no chronic complications were included. Quality of life and psychological status were evaluated using the Pediatric Quality of Life Inventory (PedsQL-C) and the Revised Children’s Anxiety and Depression Scale (RCADS). Healthy children matched for age, sex, and sociodemographic background served as controls.

**Results:**

Thirty-six patients and 41 controls participated. Children post-HSCT had significantly lower physical, psychosocial, and total quality-of-life scores (p = 0.022, 0.015, 0.006) and higher depression scores (p = 0.027). One-quarter of school-aged patients were not attending school, and one-third of families restricted exposure to crowded environments.

**Conclusion:**

Despite immune recovery, children remain vulnerable to psychosocial challenges. These findings support incorporation of routine psychological assessment and family counseling into long-term post-HSCT follow‑up.

## Introduction

Inborn Errors of Immunity (IEIs) are a rare group of genetically inherited disorders associated with significant morbidity and mortality. These conditions can present with a wide range of clinical manifestations, including increased susceptibility to severe infections, autoimmune diseases, malignancies and autoinflammatory disorders. Many affected individuals may require repeated hospitalizations and, in some cases, admission to intensive care units (ICUs) [1].

For certain types of immunodeficiencies, hematopoietic stem cell transplantation (HSCT) represents the only curative treatment option. However, patients often experience disease-related complications and undergo challenging treatment regimens before transplantation. While HSCT has yielded highly successful outcomes, treatment success depends on various factors, including the patient’s genetic diagnosis, donor compatibility, the development of graft-versus-host disease (GVHD) and the clinical condition of the patient at the time of transplantation. Furthermore, HSCT involves an extended preparatory phase and carries the risk of both short- and long-term complications, including mortality [2].

Living with a life-threatening chronic illness and undergoing a high-risk treatment such as hematopoietic stem cell transplantation (HSCT) represents an immensely challenging experience for both patients and their families. Although patients who achieve immune reconstitution after transplantation are considered immunologically cured, the psychological and emotional consequences of their disease trajectory and treatment process may persist well beyond clinical recovery[3–6]. To minimize potential confounding factors, this study included only patients who had attained complete immune recovery following transplantation and excluded those with coexisting chronic illnesses or transplantation-related complications. These children are theoretically expected to exhibit physical and psychological development comparable to that of their healthy peers. However, long-term outpatient observations have indicated that subtle differences in psychosocial adjustment may still persist despite full immunological recovery.

This study therefore aims to assess whether these experiences influence the quality of life and anxiety levels of children following HSCT by comparing them with healthy peers.

## Method

The study included children aged 8–18 years with IEIs who had achieved complete immune reconstitution following HSCT. These patients were being followed at the Pediatric Allergy and Immunology Clinics of Istanbul University-Cerrahpasa, Cam and Sakura City Hospital and Marmara University. A control group of healthy children was recruited from pediatric outpatient clinics and selected to achieve approximate matching in terms of age, sex, and sociodemographic background. Because the Pediatric Quality of Life Inventory (PedsQL) is validated for children aged ≥ 8 years, only this age group was included in the study.

The study protocol was approved by the Istanbul University-Cerrahpasa Institutional Ethics Committee (approval number:18.08.2023–755009). All participants were informed about the study procedures. Because the children were literate and over 8 years old, they signed the informed consent form along with their parents.

Patients with chronic complications related to their primary disease or transplantation—such as graft-versus-host disease (GVHD) or syndromic conditions—were excluded to minimize potential confounding factors. Only patients who were considered clinically cured after HSCT were eligible for inclusion. Demographic and clinical data were collected from hospital records, telephone interviews, and outpatient clinic visits.

T-cell immune reconstitution was defined as a CD3⁺ cell count exceeding 1,000 cells/mm^3^, a CD4⁺ cell count exceeding 500 cells/mm^3^, a phytohemagglutinin (PHA) response greater than 30% of the lower limit of the normal range or a normal stimulation index. B-cell reconstitution was defined by the recovery of serum IgA levels and independence from intravenous immunoglobulin (IVIG) therapy [7].

Psychological well-being and health-related quality of life were assessed using the Revised Children’s Anxiety and Depression Scale (RCADS) and the Pediatric Quality of Life Inventory – Child Version (PedsQL-C). Following the completion of clinical evaluation, all participants—both patients and controls—were instructed to complete the questionnaires independently in a designated quiet room within the outpatient clinic to ensure standardized conditions, minimize external distractions, and enhance the reliability of responses.

### Measures

The Revised Children’s Anxiety and Depression Scale (RCADS) is a 47-item self-report questionnaire designed for children and adolescents aged 8 to 18 years. It includes subscales measuring separation anxiety disorder, social phobia, generalized anxiety disorder, panic disorder, obsessive–compulsive disorder and major depressive disorder [8]. The validity and reliability of the Turkish version were established by Gormez et al., with a Cronbach’s alpha coefficient of 0.95, indicating excellent internal consistency [9].

The Pediatric Quality of Life Inventory – Child Version (PedsQL-C) is a 23-item questionnaire developed to assess health-related quality of life in children. Each item is rated on a 5-point Likert scale (0 to 4) based on symptom severity. The tool provides subscale scores for psychosocial and physical health, as well as a total score. Higher scores indicate better perceived quality of life [10]. In this study, validated Turkish versions of the 8–12 and 13–18 age self-report forms were used (Cronbach’s alpha values were 0.86 and 0.82, respectively). [11, 12].

### Statistical analysis

Data analysis was performed using IBM SPSS Statistics version 26.0 (IBM Corp., Armonk, NY, USA). Normality of continuous variables was assessed both visually and statistically. Categorical variables were presented as frequency (n) and percentage (%), while continuous variables were reported as median [minimum–maximum] or mean ± standard deviation (SD), depending on the distribution.

Comparisons between categorical variables were conducted using the chi-square test or Fisher’s exact test, as appropriate. Continuous variables were compared using the Student’s t-test or Mann–Whitney U test based on normality assumptions.

Spearman’s rank correlation was used to evaluate relationships between nonparametric variables, with correlation strength classified as follows: weak (rho = 0.20–0.39), moderate (0.40–0.59), strong (0.60–0.79), and very strong (≥ 0.80). Linear regression analysis was performed to identify factors associated with total health scores. Regression coefficients (B), 95% confidence intervals (CIs), R-squared values, and collinearity statistics were calculated for each predictor. A two-sided p-value of < 0.05 was considered statistically significant.

## Results

Among a total of 123 patients who had undergone HSCT for inborn errors of immunity (IEI) across the three participating centers, 58 were aged above 8 years. Of these, 3 declined participation, 5 had syndromic diagnoses associated with chronic complications, 6 had chronic graft-versus-host disease (GVHD), 5 had chronic complications related to either the primary disease or transplantation and 3 had both GVHD and other chronic complications. Consequently, 36 patients who met the inclusion criteria and 41 healthy controls were included in the final analysis.

There were no significant differences between the groups in terms of the children’s age (patients: 11.9 ± 3.2 years; controls: 12.0 ± 2.6 years, p = 0.85) or sex distribution (p = 0.26), Mothers in the control group were significantly older than those in the patient group (p = 0.006), while the difference in fathers’ age approached statistical significance (p = 0.091). Regarding psychiatric history, a significantly higher proportion of children in the patient group had a diagnosed psychiatric disorder after transplantation compared to the control group (p = 0.023). These psychiatric diagnoses included major depressive disorder (patient group n = 7) and attention deficit hyperactivity disorder (patient group n = 2, control group n = 2). Although other psychiatric variables—such as parental psychiatric history and prior psychiatric consultations for the child—were more frequently reported in the patient group, these differences did not reach statistical significance (p > 0.05). Importantly, psychiatric medication (antidepressant) use was observed only among children in the patient group (Table 1).

**Table 1:** Demographic features of the study population

	Patient Group (n = 36)	Control Group (n = 41)	P value
Age, years, mean ± SD	11.9 ± 3.2	12.0 ± 2.6	0.85
Sex, Female (n, %)	13 (36.1%)	20 (48.8%)	0.26
Mother’s Age, years	37.9 ± 5.6	41.5 ± 5.3	**0.006**
Father’s Age, years	41.8 ± 5.6	44.0 ± 5.9	0.091
Psychiatric History (n, %)			
Child’s psychiatric admission	10 (27.8%)	5 (12.2%)	0.085
Child’s psychiatric diagnosis	9 (25.0%)	2 (5.3%)	**0.023**
History of psychiatric medication use (child)	7 (20.0%)	0 (0%)	**0.004**

Genetic confirmation was available for several cases. In the SCID group, diagnoses included adenosine deaminase (ADA) deficiency (n = 2) and IL2RG deficiency (n = 1). In the combined immunodeficiency group, identified mutations included DOCK8 (n = 2), RLTPR (n = 1), FCHO1 (n = 1), Wiskott-Aldrich syndrome (WAS) (*n* = 4) and MALT1 (n = 2). Among patients with phagocyte defects, chronic granulomatous disease was diagnosed in six cases (X-linked: n = 4; autosomal recessive: n = 2), and leukocyte adhesion deficiency type 1 (LAD1) in two patients. In the immune dysregulation group, diagnoses included *LRBA* deficiency (n = 2) and primary hemophagocytic lymphohistiocytosis (HLH)-related immunodeficiencies (n = 2).

Notably, 22.2% of children in the patient group were not attending school after completion of compulsory basic education, whereas all children in the control group continued regular school attendance. In addition, approximately one-third of primary caregivers in the patient group reported perceiving crowded environments as unsafe for their children, based on responses to the questionnaire item, *“Do you consider it unsafe for your child to participate in crowded environments?”* None of the caregivers in the control group reported such concerns. Table 2 summarizes the clinical characteristics of the patient group (n = 36).

**Table 2:** The clinical characteristics of patients undergoing HSCT

Clinical Features	Patient Group (n = 36)
Age at Diagnosis	5.6 ± 7.4
Diagnosis of the patient	
Combined Immune Deficiency	17 (47.2%)
Severe Combined Immune Deficiency	7 (19.5%)
Phagocyte Defects	8 (22.2%)
Immune dysregulation	4 (11.1%)
Hospitalization before transplantation	32 (88.9%)
ICU Hospitalization before transplantation	9 (25.0%)
Severe Infection before transplantation	25 (69.4%)
Type of Donor	
MUD	16 (44.4%)
MSD	15 (41.6%)
HID	3 (8.3%)
UD	2 (5.6%)
Conditioning Regimens	
Reduced intensity	26 (72.2%)
None	7 (19.4%)
High dose	3 (8.3%)
Time from diagnosis to transplantation, months	12 (2–180)
Time since transplantation, months	81(24–156)
Hospitalization after transplantation	24 (66.7%)
ICU stay after transplantation	5 (13.9%)
Acute GVHD	7 (19.4%)
Other acute complications	5 (13.9%)
Return to School	28 (77.8%)
Avoidance of crowds	12 (33.3%)

*ICU*, Intensive Care Unit; *HID*, haploidentical donor; *MSD,* matched sibling donors; *MUD*, matched unrelated donor; *UD,* unrelated donor; *GVHD*, graft versus host disease

Children in the patient group demonstrated significantly lower scores in physical health, psychosocial health, and total health compared to controls (p = 0.022, p = 0.015, and p = 0.006, respectively). Regarding psychological symptom domains, the depression score was significantly higher in the patient group (p = 0.027). Table 3 presents a comparison of psychological assessment scores between the patient and control groups.

**Table 3:** The comparison of the psychological assessment scores

Feature	Patient Group (n = 36)	Control Group (n = 41)	p-value
RCADS			
Total Scale Score	26.0 (0.0–103.0)	20.0 (0.0–80.0)	0.10
Total Anxiety Score	21.0 (0.0–91.0)	18.0 (0.0–69.0)	0.22
Separation Anxiety	4.0 (0.0–21.0)	2.0 (0.0–10.0)	0.066
Generalized Anxiety	5.0 (0.0–15.0)	3.0 (0.0–14.0)	0.18
Panic Disorder	2.0 (0.0–20.0)	2.0 (0.0–19.0)	0.65
Social Phobia	6.0 (0.0–22.0)	7.0 (0.0–19.0)	0.72
OCD	4.0 (0.0–16.0)	2.0 (0.0–15.0)	0.074
Depression	5.0 (0.0–22.0)	3.0 (0.0–16.0)	**0.027**
**PedsQL-C**			
Physical Health	78.1 (6.3–100.0)	87.5 (56.3–100.0)	**0.022**
Psychosocial Health	83.3 (18.3–98.3)	88.3 (46.6–100.0)	**0.015**
Total Health	81.5 (14.1–95.4)	88.0 (56.5–100.0)	**0.006**

*PedsQL-C*; Pediatric quality of life inventory child versions, *RCADS;* Revised Children’s Anxiety and Depression Scale, *OCD;* obsessive compulsive disorder

Within the transplanted patient group, total PedsQL (total health score) and total RCADS (total scale score), as well as their respective subdomains, were compared between children who were attending school and those who were not. Among these comparisons, only the physical health score was significantly lower in children who were not attending school compared to those who had returned to school (*p* = 0.041). No other PedsQL or RCADS subscale differences reached statistical significance. Similarly, when comparing patients whose caregivers reported avoiding crowded environments with those who did not, no significant differences were observed in either PedsQL or RCADS scores (*p* > 0.05 for all comparisons).

In the patient group, higher anxiety and depression scores were significantly associated with lower PedsQL scores. The strongest negative correlations were observed between depression and total PedsQL (r = –0.573, *p* < 0.001), total anxiety and total PedsQL (r = –0.466, *p* < 0.001), and obsessive–compulsive symptoms and psychosocial health (r = –0.443, *p* < 0.001) (Table 4). However, when these psychological variables were entered into the linear regression model, no significant associations were found between total PedsQL scores and any of the RCADS subdomains, including generalized anxiety, separation anxiety, panic disorder, social phobia, depression, or obsessive–compulsive symptoms (all *p* > 0.05).

**Table 4:** Correlations between RCADS subscales and PedsQL scores

	1	2	3	4	5	6	7	8	9	10	11
1.RCADS-Separation Anxiety	1.00	.593**	.495**	.684**	.729**	.509**	.832**	.804**	-.450**	-.449**	-.578**
2. RCADS-Generalized Anxiety		1.00	.571**	.709**	.732**	.424**	.830**	.777**	-.174	-.403*	-.347*
3. RCADS-Panic Disorder			1.00	.602**	.476**	.532**	.703**	.718**	-.358*	-.326	-.343*
**4.** RCADS**-Social Phobia**				1.00	.699**	.558**	.890**	.880**	-.200	-.337*	-.356*
**5.** RCADS**-OCD**					1.00	.427**	.864**	.811**	-.278	-.443**	-.456**
6. RCADS-Depression						1.00	.578**	.739**	-.496**	-.491**	-.573**
**7.** RCADS**-Total Anxiety Score**							1.00	.968**	-.335*	-.404*	-.466**
**8.** RCADS**-Total Scale Score**								1.00	-.388*	-.416*	-.494**
9.PedsQL-Physical Health									1.00	.493**	.860**
10. PedsQL-Psychosocial Health										1.00	.822**
**11.** PedsQL**-Total Health**											1.00

*PedsQL-C*; Pediatric quality of life inventory child versions, *RCADS;* Revised Children’s Anxiety and Depression Scale, *OCD;* obsessive compulsive disorder

^**^ p < 0.001,* p < 0.05

The relationships between demographic and clinical variables in relation to PedsQL scores were evaluated. No significant correlations were observed between PedsQL scores and patients’ age or the number of hospitalizations before and after transplantation. However, age at diagnosis showed a negative correlation with psychological health (r = –0.488, *p* = 0.003), indicating lower psychosocial functioning among patients diagnosed later. In contrast, time since transplantation demonstrated a positive correlation with physical health (r = 0.352, *p* = 0.041).

No significant correlations were found between any demographic or clinical variables and PedsQL or RCADS scores. Variables considered clinically relevant—including patient age, age at diagnosis, time since transplantation, number of hospitalizations, and the RCADS total score—were subsequently included in a multivariable linear regression model. This analysis did not reveal any statistically significant predictors of PedsQL scores, although a weak positive association was observed between the number of hospitalizations and total health score (*p* = 0.033).

## Discussion

Hematopoietic stem cell transplantation is a highly specialized treatment that enables children with potentially fatal or organ-damaging diseases, such as IEIs, to reintegrate into society as healthy individuals. For these patients—who successfully undergo this challenging procedure and remain free of chronic complications—achieving a quality of life comparable to their peers is crucial. This requires comprehensive, multifaceted evaluation and identification of modifiable factors that may influence their quality of life.

Patients with IEIs face significant challenges during treatment of the primary disease and throughout the transplantation period, including prolonged hospitalizations, ICU admissions, immobilization and social isolation [13, 14]. In our study, we aim to assess whether this arduous process impacts patients’ quality of life and psychological well-being, even after clinical recovery, by comparing them with healthy controls.

We found that the quality of life of patients was low both physically and psychosocially after recovery. Consistent with the literature, a study assessing adult patients who underwent HSCT for IEIs during childhood also reported lower physical quality of life scores compared to controls [15]. Despite immune reconstitution, 33.3% of primary caregivers believe exposure to crowded environments is harmful, and 22.7% of school-aged patients do not attend school despite having no physical barriers to do so. School attendance may represent an important functional indicator of post-transplant reintegration and long-term adaptation. Although the underlying reasons for school non-attendance were not systematically assessed in this study, this finding highlights the importance of evaluating school reintegration during long-term follow-up after HSCT. Protective parenting attitudes were also not formally assessed; however, such behaviors may unintentionally limit children’s social participation and potentially affect quality of life. During post-transplant follow-up visits, clinicians often focus primarily on the physical health of the patients. Therefore, return to school should be actively assessed during long-term follow-up, and families may benefit from structured guidance regarding safe reintegration into school and social environments. In particular, promoting engagement in age-appropriate physical or recreational activities may contribute positively to both physical recovery and psychosocial well-being.

In our subgroup analyses, the physical health score was significantly lower in children who were not attending school compared to those who had returned to school (p = 0.041), whereas no significant differences were observed in psychosocial or psychological outcomes. This finding may suggest a relationship between school participation and perceived physical well-being after HSCT. However, the lack of broader statistical significance may be partly attributable to the limited sample size, which could have reduced the power to detect subtle group differences.

A study in the literature examining the quality of life in patients with SCID after HSCT found that those continuing immunoglobulin replacement treatment (IgRT) had lower quality of life scores, while patients who had completed IgRT showed no significant difference in quality of life compared to healthy controls [16]. In SCID, patients are typically diagnosed and transplanted at a much younger age due to the rapid progression of the disease, resulting in a shorter duration of illness before transplantation. This early intervention may lessen the impact of the primary disease on quality of life [17–19]. Similarly, a study evaluating patients with chronic granulomatous disease reported that transplanted individuals had improved quality of life comparable to that of the healthy population[20]. In our study, however, due to the limited sample size, we did not perform subgroup comparisons of quality of life across different disease categories. Our patient group is more heterogeneous; some patients experience prolonged exposure to the primary disease either due to delayed diagnosis or extended time spent searching for a suitable donor. This variability may influence their quality of life.

Within this cohort, a modest negative correlation was observed between age at diagnosis and psychological health; however, this relationship did not retain statistical significance in the multivariable regression analysis. This trend may indicate a potential association between earlier diagnosis and more favorable psychological outcomes, although causality cannot be inferred from the present data.

Depression scores were significantly higher in the patient group compared with healthy controls, and psychiatric referrals and medication use were also more frequent among these patients. These findings suggest that psychological difficulties may persist even after immune recovery. Consistent with this, higher anxiety and depression scores were correlated with lower health-related quality of life. However, these associations did not remain statistically significant in the multivariable analysis, possibly reflecting the small sample size and the interrelated nature of psychological variables. Incorporating routine psychological assessment into post-transplant follow-up could therefore facilitate the early identification and management of emotional or behavioral concerns, potentially supporting better long-term psychosocial outcomes.

Nicholson et al. reported that anxiety levels in childhood HSCT recipients were significantly higher in adulthood compared to the general population [15]. In contrast, our study found no significant differences between the patient and control groups in generalized anxiety, panic, social phobia, total anxiety, or total scale scores. This discrepancy may be explained by adults’ greater awareness and knowledge of health-related issues, as well as differing expectations regarding their health compared to children [21].

Several studies have evaluated quality of life and anxiety in patients who underwent HSCT during childhood; however, these patient groups were often heterogeneous, including individuals with malignancies, metabolic diseases, and IEIs [22–24]. The present study is among the few that specifically examine quality of life in children transplanted due to IEIs without chronic complications. In contrast to IEIs, malignancies involve distinct challenges such as disease-related complications, treatment toxicities and risks of relapse, all of which can significantly influence post-recovery anxiety and quality of life. Furthermore, treatment protocols during transplantation may vary between these conditions. For these reasons, evaluating these diverse patient populations together may not provide an accurate representation of their unique post-transplant experiences [25, 26].

Patients with various types of IEIs were included and categorized into four underlying disease groups. Although all are classified under IEIs, these conditions differ in clinical course and transplantation-related complication risks, which may influence quality of life and anxiety outcomes after recovery. [27, 28]. The heterogeneity of the patient group and the varying post-transplantation periods represent limitations of this study. Additionally, this cross-sectional design restricts the ability to monitor psychological states over time. Prospective studies with larger, more homogeneous cohorts would allow evaluation of changes in psychological status pre- and post-transplant. However, pre-transplant psychological assessments are often not feasible for patients transplanted at a very young age. It should also be noted that the control group was selected from children attending pediatric outpatient clinics for routine visits and without any chronic illness, whereas the patient cohort consisted of families from different regions of the country. Therefore, a perfect sociodemographic match between groups could not be fully achieved, which may have introduced potential bias. In particular, maternal age differed between the groups, and parental age-related sociodemographic differences may have influenced psychosocial outcomes and quality-of-life measures. The lack of statistically significant predictors may be partly explained by the small sample size and the relatively homogeneous nature of our cohort, as all participants were clinically stable and free of chronic complications. Nevertheless, we included key clinical variables in the regression model to account for potential confounding effects and to explore possible trends, which could guide future larger-scale studies.

This study addresses an underexplored aspect of post-transplant outcomes in pediatric IEI patients who have achieved immune recovery. Post-transplant follow-up in such cases often prioritizes physical health and graft outcomes, while psychosocial dimensions may be overlooked. By examining a clinically homogeneous group without chronic complications, the study demonstrates that psychosocial challenges may persist despite successful medical recovery. These findings emphasize the importance of integrating psychological and social evaluation into long-term care for this rare patient population.

## Conclusion

Even after achieving immune recovery following HSCT for IEIs, children may not fully regain age-appropriate physical and psychosocial functioning. A considerable proportion of families continue to express concerns about social participation, indicating persistent emotional or behavioral factors influencing post-transplant adaptation. Incorporating structured psychosocial assessment and family counseling into post-transplant follow-up may help support reintegration and improve overall well-being. Future multicenter studies with larger and more diverse cohorts are needed to validate these findings and to further elucidate the long-term psychosocial outcomes in this population.

## Data Availability

The data underlying this article are available in the article and in its online supplementary material.

## Abbreviations

CI: Confidence Interval
GVHD: Graft-versus-host disease
HSCT: Hematopoietic stem cell transplantation
IEI: Inborn errors of immunity
PedsQL-C: Pediatric Quality of Life InventoryChild Version
RCADS: Revised Children’s Anxiety and Depression Scale
